# Lobaric acid prevents the adverse effects of tetramethrin on the estrous cycle of female albino Wistar rats

**DOI:** 10.1371/journal.pone.0269983

**Published:** 2022-07-01

**Authors:** Ha Thi Nguyen, Haritha Polimati, Satya Sowbhagya Priya Annam, Emmanuel Okello, Quynh-Mai Thai, Thien-Y. Vu, Vinay Bharadwaj Tatipamula

**Affiliations:** 1 Center for Molecular Biology, College of Medicine and Pharmacy, Duy Tan University, Da Nang, Vietnam; 2 Pharmacology Department, AU College of Pharmaceutical Sciences, Andhra University, Visakhapatnam, Andhra Pradesh, India; 3 Veterinary Medicine Teaching and Research Center, School of Veterinary Medicine, University of California, Davis, Tulare, CA, United States of America; 4 Faculty of Pharmacy, Ton Duc Thang University, Ho Chi Minh City, Vietnam; Colorado State University, UNITED STATES

## Abstract

Tetramethrin (Tm) is a commonly used pesticide that has been reported to exert estrogen-antagonistic effects selectively on female rats. The present study was undertaken to assess the protective role of lobaric acid (La) on estrous cycle in Tm-treated female Wistar rats. Female rats were exposed to Tm (50 mg/kg b.w/day) only or in combination with La at low (50 mg/kg b.w/day) or high (100 mg/kg b.w/day) dose for 30 days. The results showed that Tm altered the estrous cycle of female rats by decreasing the levels of luteinizing hormone, follicular-stimulating hormone, progesterone, estrone, and estradiol while increasing testosterone level. The morphology of vaginal smears of Tm-treated female rats showed the presence of abnormal cells and/or structures at different phases of estrus cycle. Strikingly, in (Tm + La)-treated rats, all the observed adverse effects of Tm on the hormonal parameters, cell morphology, and the length of each phase of estrous cycle were significantly diminished in a dose-dependent manner. The docking results showed that La competes with Tm for Gonadotropin-Releasing Hormone (GnRH) receptor, thereby reducing the toxicity of Tm but did not cancel the response of GnRH receptor completely. In conclusion, our results designated that La could be used as a potential candidate in the management of insecticide-induced alterations of the reproductive cycle of rodents.

## Introduction

Insecticides are widely used in agriculture and animal husbandry practices to increase the crop yield. However, the widespread usage of insecticides in agriculture, animal husbandry, and public health may lead to environmental pollution and persistence of their residues in the food chain, which may consequently result in an accidental exposure of non-targeted organisms [1–3] and unexpected side-effects on their reproductive endocrinology [4, 5]. Recently, the discovery and usage of new insecticide groups with higher toxicity and faster diffusion into the surrounding environments has raised significant concerns about their potential negative impacts on the health of all living organisms, including human beings [6].

Tetramethrin (Tm), a synthetic derivatives of type I pyrethroid (pyrethrins), is commonly used in either aerosol formulation (0.1–0.25%), aqueous sprays (0.1–0.25%), emulsifiable concentrates (2.5%), or mosquito coils (~0.54%) for the control of indoor pest such as wasps, hornets, cockroaches, ants, fleas, and mosquitoes [7]. The active ingredient in Tm is either 1,3,4,5,6,7-hexahydro-1,3-dioxo-2H-isoindol-2-yl)methyl-2,2-dimethyl-3-(2-methyl-1-propenyl)cyclopropanecarboxylate or 3,4,5,6-tetrahydrophthalimidomethyl-(1*RS*)-cis-trans-chrysanthemate, and is generally marketed with trade names of Duracide, Neo-Pynamin, and Neo-Pynamin Forte [8]. Tm has been reported to exert its estrogen-antagonistic effects including altered sexual cycle and selective inhibition of ovulation in female rats [5, 9], possibly by its specific interaction with the endocrine system of these animals [5, 10–12].

Traditionally, decoction from lichens, namely *Stereocaulon vulcani* (Bory) Ach. (Réunion folk name: fleur de roche/fleur galet), *Usnea diffracta* Vain. (China folk name: huán liè sōng luó), *Usnea zahlbruckneri* (China folk name: jīn sīdài), and *Usnea trichodeoides* Vain. (China folk name: cūpí sōng luó) have been used to treat menstrual disorders, vaginal discharge and swelling of female genitalia [13]. These findings suggested the presence of compound(s) with the protective effects against the above-mentioned disorders, possibly being a single or a combination of two or more major chemical substances of the decoction. Notably, lobaric acid (La) obtained from *Stereocaulon* and *Usnea* lichen genus has been reported for its multiple bioactivities such as anti-inflammatory and anti-gout [14], antioxidant [15], anticancer [16], and enzyme inhibitory activities [17]; however, its protective effects on the estrous cycles remained poorly understood. In the current study, we aimed to investigate the ability of La to prevent the negative effects of Tm on reproductive hormone levels and estrous cycle in female albino Wistar rats.

## Materials and methods

### Chemicals

**Fig 1:**
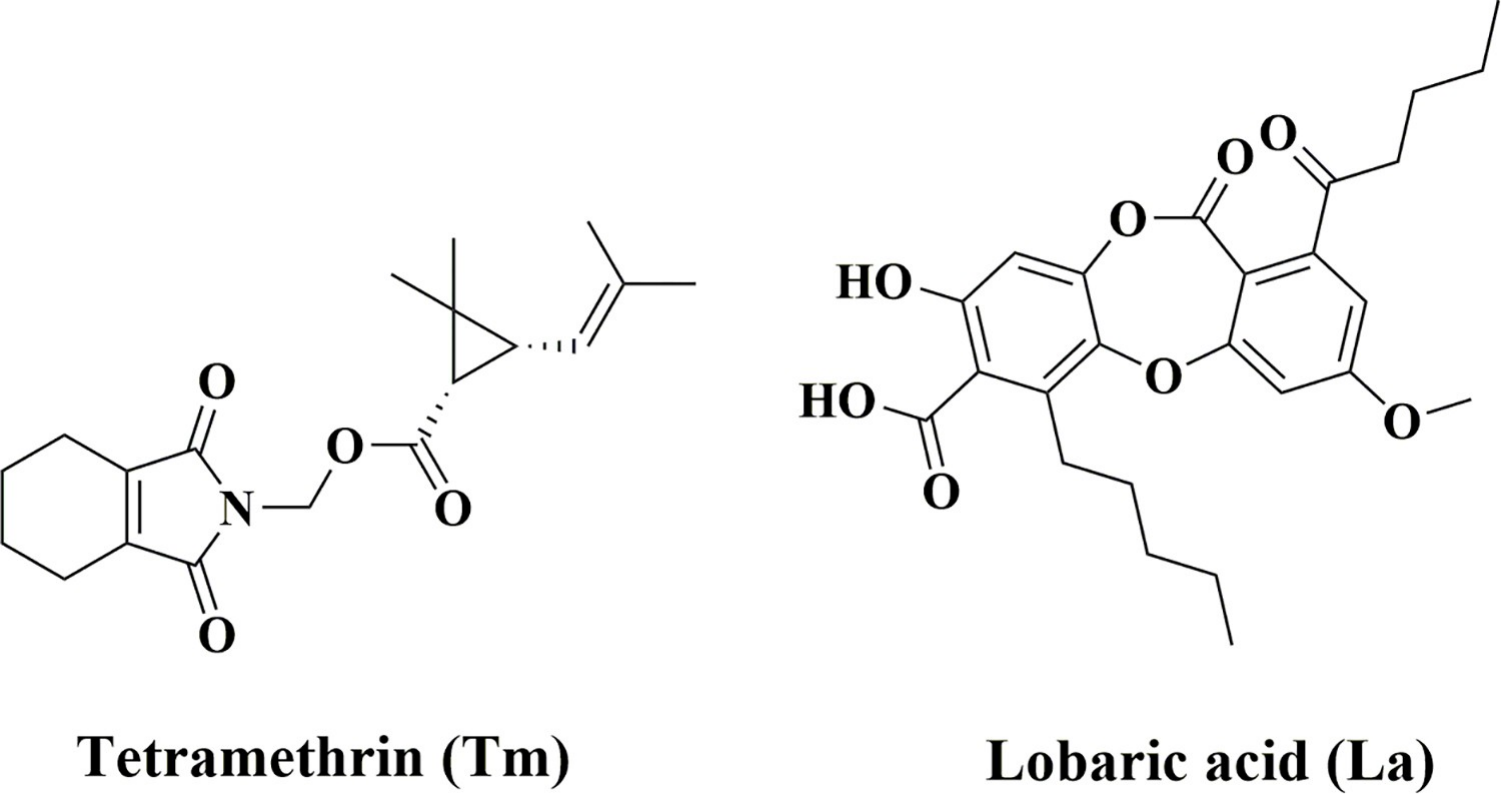
Chemical representation of tetramethrin (insecticide) and lobaric acid (lichen metabolite).

Tm was procured from Daga Global Chemicals FZCO (Gujarat, India), and La (above 95% pure) was previously isolated from *Usnea subfloridana* Stirton by our group [14]. All the other chemicals used in this study are of analytical grade. The chemical structures of Tm and La are shown in Fig 1 below.

### Experimental animals

A total of 48 female Wistar rats (4-months old and weighing 180–200 g) were used in this study. The animals were acclimatized to the experimental conditions for one week before conducting the study. The animals were then housed in solid bottom polypropylene cages inside the lab animal house at Andhra University, with a controlled room temperature maintained at 20–22°C throughout the course of the experiment. Rice husk was used as the bedding material. All the animals were provided with standard pellet diet and water *ad libitum* throughout the experimental period. The experiment was conducted according to the guidelines and with prior approval of the Institutional Animal Ethics Committee of Andhra University, Visakhapatnam, India (approval number: 516/PO/c/01/IAEC/951, dated 19 September 2020).

### Experimental design

The female Wistar rats were randomly and equally divided into four groups of twelve rats each (*n* = 12). The first group served as normal control (NC) group that was fed with 0.5% sodium carboxymethyl cellulose only; the second group received only Tm at 50 mg/kg b.w/day (Tm group); the third group was given both Tm (50 mg/kg b.w/day) and La at low dose (50 mg/kg b.w/day) (Tm + La-L); and the fourth group was treated with both Tm (50 mg/kg b.w/day) and La at high dose (100 mg/kg b.w/day) (Tm + La-H). The test compound was administered for 30 consecutive days between 9–10 am by oral gavage (1.0 ± 0.1 ml (low dose) and 2.0 ± 0.2 ml (high dose)).

### Evaluation of estrous cycle and organ weight

Up to seven consecutive estrous cycles were monitored for all four groups by performing vaginal smear test (taken twice daily at 8 am and 5 pm) as previously described [18, 19]. Briefly, the method consists of flushing of cells from the vaginal lining by gently inserting the tip of a soft plastic pipette containing approximately 0.2 ml of 0.9% saline into rat vaginal orifice to a depth of 2–5 mm and then saline solution was gently flushed into the vagina and taken back into the pipette from 2 to 3 times. Then a small amount of the cell suspension was dropped onto a labelled clean glass slide. The slides were then stained with Giemsa and observed under a light microscope (ESAW, India) and the stage of estrous cycle was defined based on the types and the proportions of the cells in the smear [20]. The length of each estrous cycle and the relative lengths of four stages within estrous cycle was calculated as previously reported [21].

At the end of the study, after collecting blood sample from retro orbital plexus, animals were sacrificed by cervical dislocation after a mild anesthesia. Ovaries and uterus from the control and treated animals were collected and weighed.

### Hormone assessment

On experimental days 15 and 30, approximately 2 ml of blood was collected from each rat from retro orbital plexus through capillary tube into a clot promoting vacutainers and allowed to clot for 3 to 4 h. The tube was then centrifuged at 2,000 x g for 10 min and serum was transferred to a new labelled sterile Eppendorf tube and stored at -20°C. Later, luteinizing hormone (LH), follicle stimulating hormone (FSH), testosterone (T), progesterone (P4), estrone (E1), and estradiol (E2) in these serum samples were quantified by using corresponding ELISA kits.

### Docking and theoretical binding energy estimation methods

The crystal structure of the Gonadotropin-Releasing Hormone (GnRH) receptor (PDB 7BR3) was downloaded and prepared by the Protein Preparation Wizard of Maestro software (Schrödinger Release 2020–3). A general procedure was used to remove water, assign bond orders, optimize the H-bond, generate Het states using Epik, and minimize the protein with OPLS3e force field [22]. The 7BR3 protein contains the small-molecule drug elagolix. The centroid of elagolix was used to establish the grid box for docking jobs with 20 Å in three dimensions.

Next, two structures of La (PubChem CID 73157) and the synthetic insecticide Tm (PubChem CID 83975) were imported and prepared by Ligprep [23] to obtain possible ionization states at physiological pH = 7.0 ± 2.0. La, Tm and the native ligand of crystallized protein were docked to the grid box by using Glide [24] SP (standard precision). The SP results were refined with XP (extra precision) before being used to calculate the free energy by MM-GBSA [25] (Molecular Mechanics/Generalized Born and Surface Area) method with the VSGB solvation model and OPLS_2005 force field. Traditionally, the binding energy value indicates the affinity between a ligand and a protein. The more negative this energy is, the stronger affinity and the better inhibitory ability a ligand has. In a special case where two ligands have the same binding energy but have different levels of competition, more in-depth kinetic analyses such as non-equilibrium MD simulations are required.

### Pulling pathway

After examining free energies of the ligands in the binding site, a molecular kinetic procedure monitoring the separation of the ligands from the protein was carried out. The proper pulling pathways were determined by using the same procedure described previously in Caver 2.1 [26, 27], MOLE 2.0 [28], and MolAxis [29]. Firstly, the complexes were rotated until the pulling direction was oriented along the Z-axis utilizing the PyMOL package [30]. Consequently, the pulling vector was averaged from these predicted pathway vectors and the external harmonic force can be applied to pull the ligand out of the binding site along with the Z orientation [31].

### Non-equilibrium MD simulations

The external force given by Eq (1), applied on the center of mass of the ligands along with the Z orientation, was used to dissociate the ligands in the isobaric-isothermal ensemble. Consequently, the work of an external force was given by the formula (2).


F=k(vt‐z) (1)

$$W=v\int_{0}^{t}F(t)\;dt$$ (2)

Where k is the spring constant of the cantilever

v is pulling speed, and

z is the displacement of the inhibitor center of mass from its initial position.

From the binding pose of each protein-ligand complex, the ligands were pulled out of the GnRH receptor binding site after a short position restrained simulation. Eight different independent trajectories with random velocity were performed for each complex. The external force is recorded every 0.02 ps. The work of pulling force *W* was calculated through Eq (1). The average of external work *W* was averaged from eight independent non-equilibrium MD simulations.

## Results

### Organ weights

**Fig 2:**
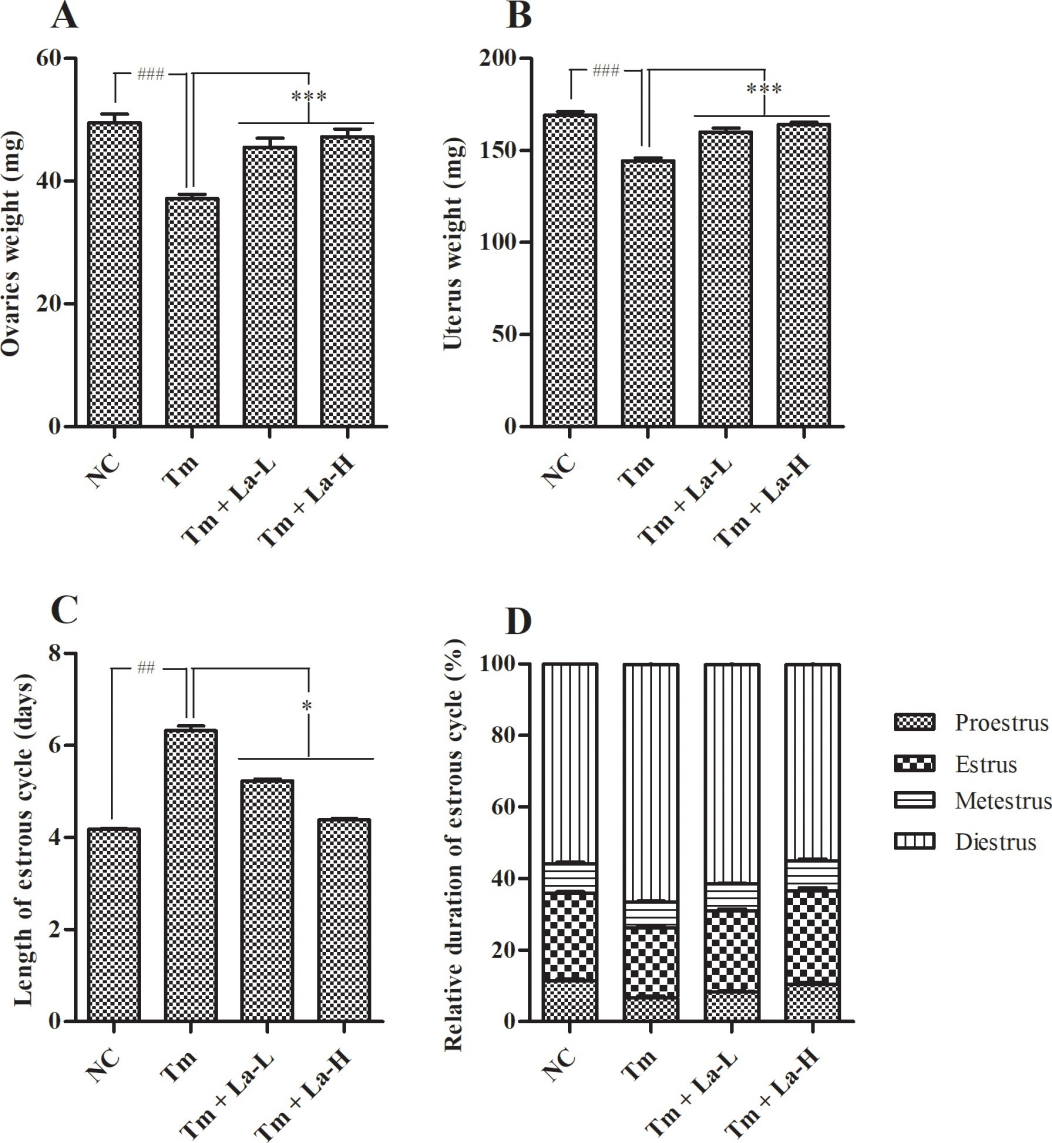
Effects of Tm and La on (A) Weight of ovaries, (B) Weight of uterus, (C) Length of estrous cycle, and (D) Relative duration of estrous cycle at day 30. Values are presented as mean ± SEM. Significant difference at ^##^*P* < 0.01, ^###^*P* < 0.001 when compared with control group and *P < 0.05, ***P < 0.001 when compared with Tm group using ANOVA followed by Dunnett’s test for analysis. SEM: standard error of the mean; NC: normal control; Tm: tetramethrin 50 mg/kg b.w; Tm + La-L: tetramethrin 50 mg/kg b.w + lobaric acid 50 mg/kg b.w; Tm + La-H: tetramethrin 50 mg/kg b.w + lobaric acid 100 mg/kg b.w.

After 30 days of experiment, all rats were sacrificed and their ovaries and uterus were collected and weighted. There was a significant decrease in the weight of both ovaries (37.17 ± 0.72 mg) and uterus (144.42 ± 1.62 mg) of Tm-treated rats as compared to that of the control rats (ovary: 49. 50 ± 1.45 mg and uterus: 169.25 ± 1.96 mg) (*P* < 0.001). Notably, La (at both low and high dose) significantly (*P* < 0.001) inhibited the decrease in weight of ovaries (45.50 ± 1.51 and 47.25 ± 1.29 mg) and uterus (160.08 ± 2.19 and 164.25 ± 1.14 mg) of the Tm-treated female rats in a dose-dependent manner (Fig 2A and 2B).

### Length of estrous cycle

Tm-treated female rats showed a drastic increase in the length of estrous cycle with a mean length of 6.32 ± 0.10 days as compared to the control group (4.18 ± 0.02 days) (*P* < 0.01). Both low and high dose of La prevented the prolonged estrous cycle of the Tm-treated female rats with the mean length of estrous cycle found to be 5.23 ± 0.04 and 4.38 ± 0.03 days, respectively (*P* < 0.05) (Fig 2C).

### Average length of each phase in estrous cycle

To assess the possible toxicity of Tm on ovarian system, we analyzed the phases of estrous cycle among the different treatment groups. Our results did not reveal any significant difference in the average length of proestrus, estrus, and metestrus phase between the control, Tm-treated and (Tm + La)-treated groups. In contrast, the length of diestrus phase found to be substantially increased in Tm-exposed female rats (4.20 ± 0.11 days) as compared to that of the control group (2.33 ± 0.03 days). Remarkably, this effect was abolished in La-treated group where the average length of diestrus phase in (Tm + La-L)- and (Tm + La-H)-treated rats found to be 3.20 ± 0.09 (*P* < 0.05) and 2.40 ± 0.06 days (*P* < 0.001), respectively (Fig 2D).

### Assessment of hormones

The effect of the Tm and La on hormone levels at day 15^th^ and 30^th^ was examined.

#### Follicular stimulating hormone and luteinizing hormone

**Fig 3:**
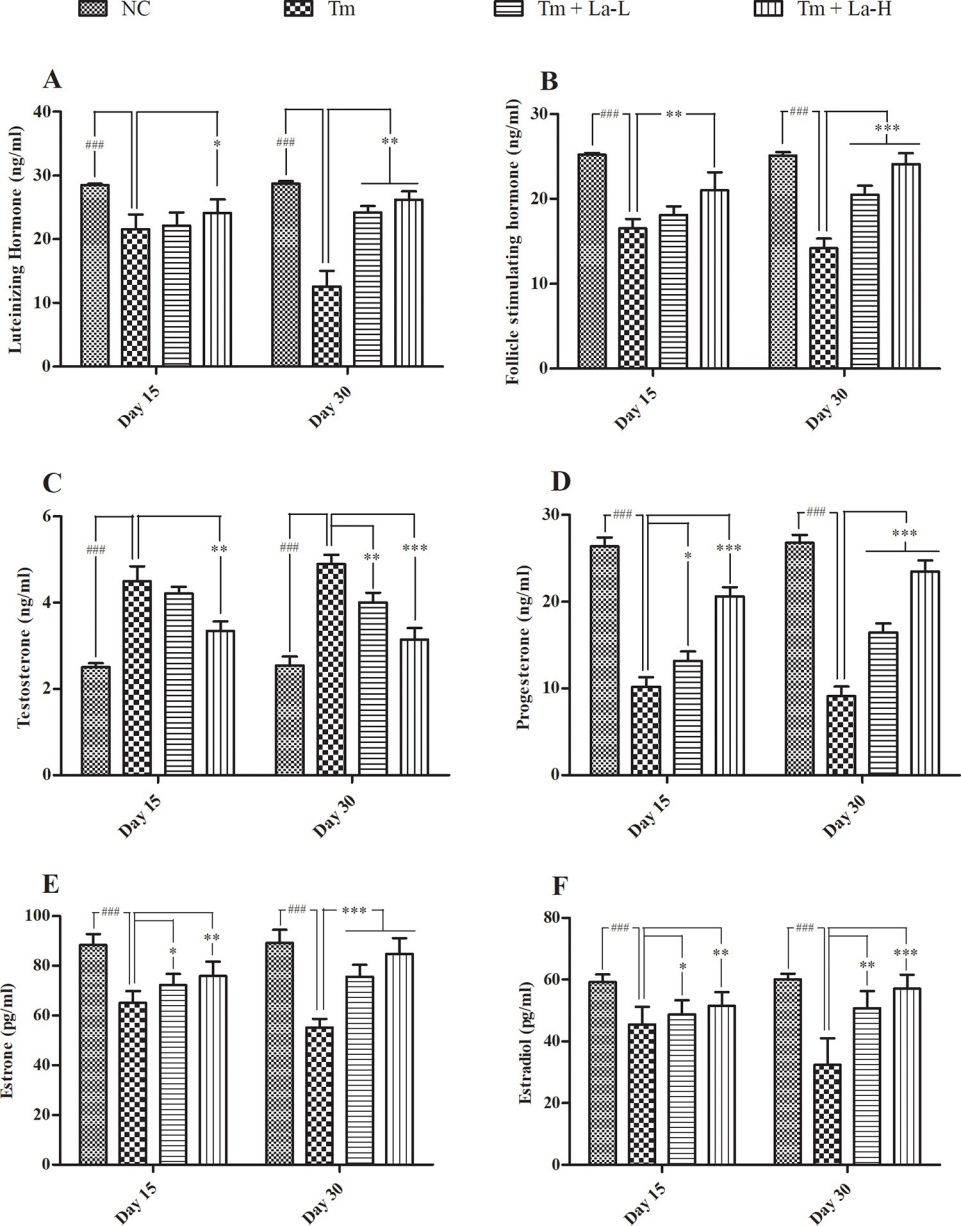
Effects of Tm and La on (A) Luteinizing hormone, (B) Follicle stimulating hormone, (C) Testosterone, (D) Progesterone, (E) Estrone, and (F) estradiol in female rats. Data are presented as mean ± SEM of 12 replicates. Significant difference at *P < 0.05, **P < 0.01, ***P < 0.001 level when compared with Tm group and ^###^*P* < 0.001 when compared with control group using ANOVA followed by Dunnett’s test for analysis. Tm: tetramethrin, La: lobaric acid, SEM: standard error of the mean; NC: normal control; Tm: tetramethrin 50 mg/kg b.w; Tm + La-L: tetramethrin 50 mg/kg b.w + lobaric acid 50 mg/kg b.w; Tm + La-H: tetramethrin 50 mg/kg b.w + lobaric acid 100 mg/kg b.w.

At both intervals (day 15 and day 30), the levels of LH and FSH were significantly decreased in Tm-exposed group as compared to that of the control group (*P* < 0.001). These effects, however, were eliminated in (Tm + La)-treated rats. At day 15^th^, only the (Tm + La-H)-treated group showed a significant difference in both LH and FSH hormone levels compared to the Tm-treated group (*P* < 0.05), while there was no significant difference in the LH and FSH levels between (Tm + La-L)-treated group and the Tm-treated group (Fig 3A and 3B). At day 30^th^, both the (Tm + La-H)- and (Tm + La-L)-treated groups showed significant difference in the levels of LH (*P* < 0.01) and FHS (*P* < 0.001) as compared to Tm-treated group. Noticeably, for the (Tm + La-H)-treated rats, the levels of LH (26.22 ± 1.31 ng/ml) and FHS (24.10 ± 1.30 ng/ml) were almost equivalent levels of the control group (LH: 28.70 ± 0.40 ng/ml and FSH: 25.10 ± 0.42 ng/ml) (Fig 3A and 3B).

#### Testosterone and progesterone

In contrast to all investigated hormones in this study, Testosterone (T) was significantly higher in Tm-treated group at both day 15 and day 30 (4.50 ± 0.35 and 4.90 ± 0.21 ng/ml) as compared to the control group (2.50 ± 0.10 and 2.55 ± 0.20 ng/ml) (*P* < 0.001) (Fig 3C). In (Tm + La)-treated groups, however, the level of T was significantly suppressed at both time intervals for the (Tm + La-H) group (day 15: 3.35 ± 0.22 ng/ml, *P* < 0.01 and day 30: 3.14 ± 0.27 ng/ml, *P* < 0.001), while the suppression was only observed at day 30^th^ (4.01 ± 0.22 ng/ml, *P* < 0.01) for the (Tm + La-L) group (Fig 3C).

A significant decrease in the P4 levels was observed at both time points in the Tm-treated female rats (10.20 ± 1.11 and 9.10 ± 1.12 ng/ml) as compared to the control ones (26.40 ± 1.02 and 26.80 ± 0.90 ng/ml) (*P* < 0.001). The (Tm + La-H) group showed a significantly higher P4 level (23.47 ± 1.30 ng/ml) as compared to the Tm group (9.10 ± 1.12 ng/ml), which was almost equivalent to that of the control group (26.80 ± 0.90 ng/ml) at day 30^th^ (Fig 3D).

#### Estrone and estradiol

A significant decrease in E1 and E2 levels was detected in Tm-treated group in comparison to the control group at both time points (*P* < 0.001) (Fig 3E and 3F). These effects, however, were also reduced in (Tm + La) groups. At day 30, the E1 levels of the (Tm + La-L) and (Tm + La-H) group were found to be 75.60 ± 4.78 and 84.80 ± 6.35 pg/ml, respectively, which were similar to that of the control group (89.20 ± 5.37 pg/ml) and substantially higher than that of the Tm-treated group (55.20 ± 3.47 pg/ml) (Fig 3E). Similarly, the E2 levels were also retained in (Tm + La) groups. Particularly, the levels of E2 in (Tm + La-L) and (Tm + La-H) at day 30 were recorded at 50.82 ± 5.45 and 57.02 ± 4.57 pg/ml, respectively, which were significantly higher than that of the Tm group (32.46 ± 8.54 pg/ml) and similar to that of the control group (60.10 ± 1.86 pg/ml) (Fig 3F). Taken together, our data revealed that La significantly maintained the levels of reproductive hormones in (Tm + La)-exposed rats to a similar level of the control group in a dose- and time-dependent manner.

### Vaginal cytology

**Fig 4:**
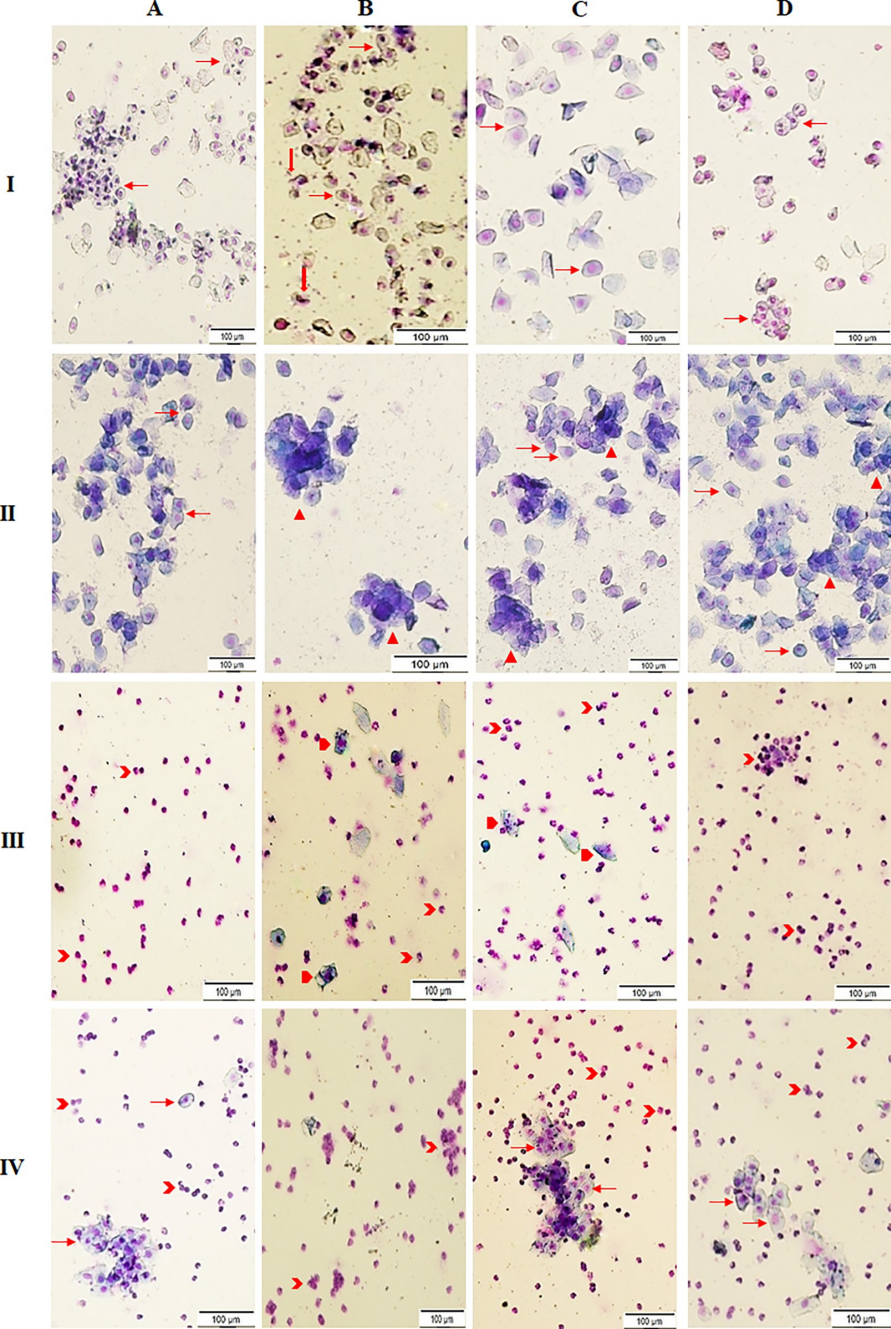
Photomicrograph of vaginal smears of the last examined estrous cycle of female Wistar rats (Scale 100 μm). (I) Proestrus phase, (II) Estrus phase, (III) Metestrus phase, (IV) Diestrus phase. (A) normal control; (B) tetramethrin 50 mg/kg b.w; (C) tetramethrin 50 mg/kg b.w + lobaric acid 50 mg/kg b.w; (D) tetramethrin 50 mg/kg b.w + lobaric acid 100 mg/kg b.w. NEC (red right arrow): nucleated epithelial cells; CSEC (red down arrow): cornified squamous epithelial cells; rEC (red isosceles triangle): ruptured epithelial cells; rNEC (red pentagon): ruptured nucleated epithelial cells; L (red chevron): leucocytes.

In general, the different stages of estrous cycle are characterized by presence of various type of cells, namely nucleated epithelial cells, anucleated cornified epithelial cells, and leucocytes in vaginal smears. The staining results of vaginal smears of the control and Tm-treated rats at four different stages of estrous cycle were presented in Fig 4. The vaginal smears of the control rats in proestrus comprised almost extensively of oval nucleated epithelial cells (NEC) in form of cohesive (grape) clusters interspersed with few cornified squamous epithelial cells (CSEC) (Fig 4-I.A) while that of the Tm-treated rats revealed ruptured epithelial cells (rEC), indicating the negative impact of Tm on the cells (Fig 4-I.B). The rEC, however, were not seen in two (Tm + La) groups (Fig 4-I.C and 4-I.D). Especially, in the (Tm + La-H)-treated group, and NEC were found in clumps similar to that of the control group (Fig 4-I.D).

In the estrus phase, smears of the control rats contained mostly NEC and CSEC arranged in loose sheets (Fig 4-II.A) whereas, cells of the Tm-treated rats formed clumps (Fig 4-II.B). These clumps were diminished in the (Tm + La)-treated groups in a dose-dependent manner (Fig 4-II.C and 4-II.D).

In the metestrus phase, smears of the control rats comprised extensively of leucocytes (Fig 4-III.A) while Tm-treated rats also showed the ruptured NEC (rNEC) and leucocytes with abnormal phenotype (Fig 4-III.B). The side effects observed in Tm-treated rats were lessen in the (Tm + La)-treated rats in a dose-dependent manner with few rNEC in (Tm + La-L) and non rNEC in (Tm + La-H) groups (Fig 4-III.C and 4-III.D). Especially, the smears of the (Tm + La-H) group comprise clumps of leucocytes, a typical feature of metestrus phase (Fig 4-III.D).

In the diestrus phase, a small clumps of NEC along with neutrophils were observed in smears of control rats indicating the occurrence of proestrus in the next day (Fig 4-IV.A) while an extensive number of leucocytes with no NEC in the smears of Tm-treated rats (Fig 4-IV.B) indicating the hormonal imbalance caused by oxidative stress effects of insecticide (Tm). The appearance of small clumps of NEC along with leucocytes in (Tm + La) groups (Fig 4-IV.C and 4-IV.D) indicates regularized estrous cycle due to antioxidant properties of La, which may also be resulted in prevention of oxidative damage to ovaries and production of hormones in appropriate quantities.

### Docking studies

#### Theoretical binding energies estimations

**Fig 5:**
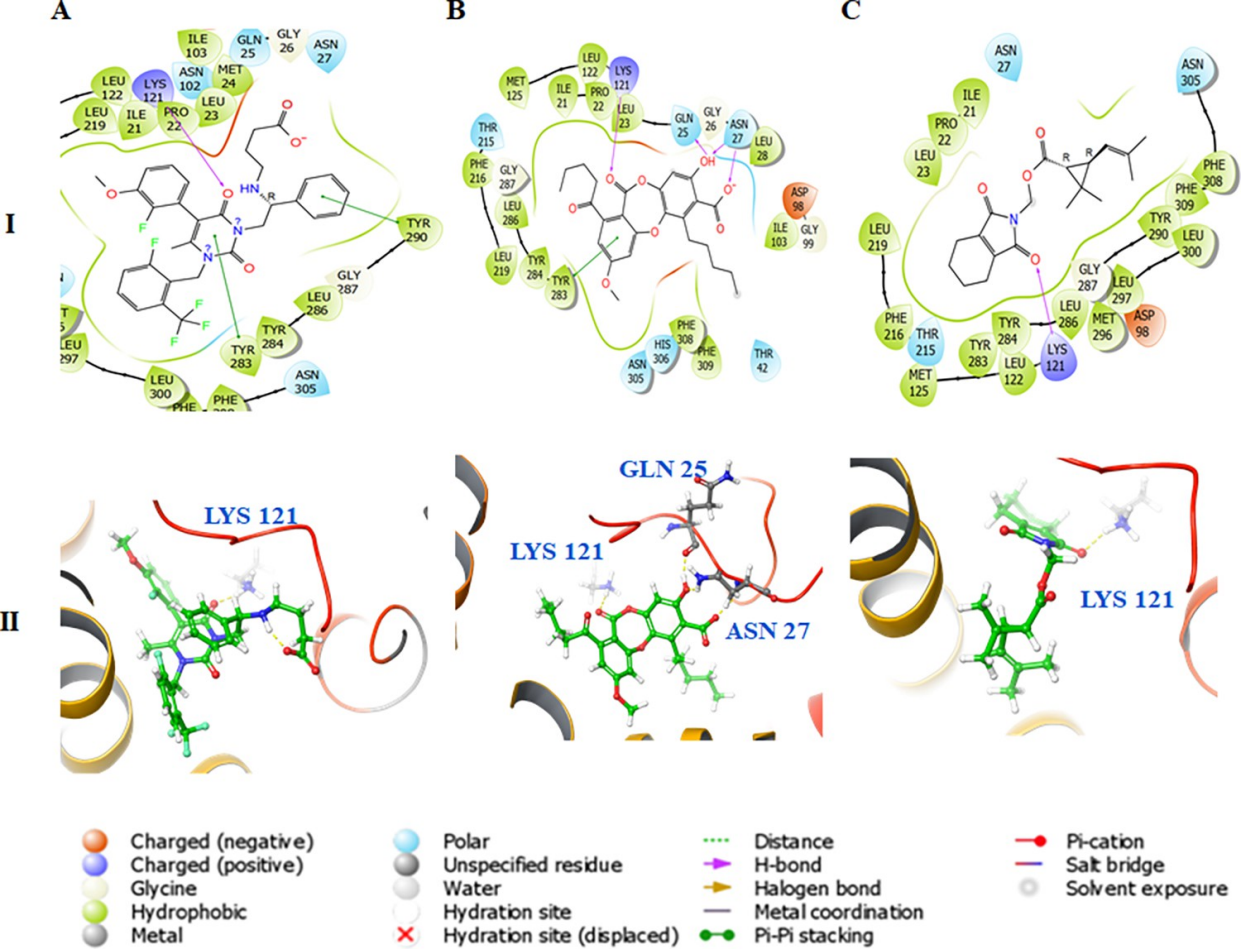
(I) 2D and (II) 3D binding pose presentations of (A) Elagolix, (B) Lobaric acid, and (C) Tetramethrin with gonadotropin-releasing hormone receptors.

The RMSD between the crystal and redocked structures of the native ligand elagolix is 0.80Å, indicating a good and reliable binding mode of docking methods. In term of energy, the elagolix drug had the lowest theoretical free energy at -112.4 kcal/mol while the La and Tm had higher free energy values at -84.4 and -84.6 kcal/mol, respectively (Table 1). All three ligands formed one H-bond with the key residue, Lys121 of the GnRH receptor, while La also formed two additional H-bonds with Gln25 and Asn27 (Fig 5). Moreover, the hydrophobic interaction contributes largely to the stabilization of the binding mode of elagolix with two π-π stacking with Tyr283 and Tyr290 while La has one π-π stacking with Tyr283 of GnRH receptor.

**Table 1:** The XP docking scores, MM-GBSA binding free energies estimations (kcal/mol), steered molecular dynamics (SMD) analysis of lobaric acid, tetramethrin, and elagolix native ligand.

Compounds	XP docking scores (kcal/mol)	MM-GBSA free energy estimation (kcal/mol)	No of H-bonds	Residues	SMD Analysis			
					Fmax (pN)	Error	W (kcal/mol)	Error
**Lobaric acid**	-9.1	-84.4	4	Lys121, Gln25, Asn27	1686.9	70.0	277.5	17.6
**Tetramethrin**	-7.8	-84.6	1	Lys121	1094.7	41.9	157.7	8.0
**Elagolix**	-14.4	-112.4	1	Lys121	2399.9	67.3	491.3	22.2

#### Non-equilibrium MD simulations

**Fig 6:**
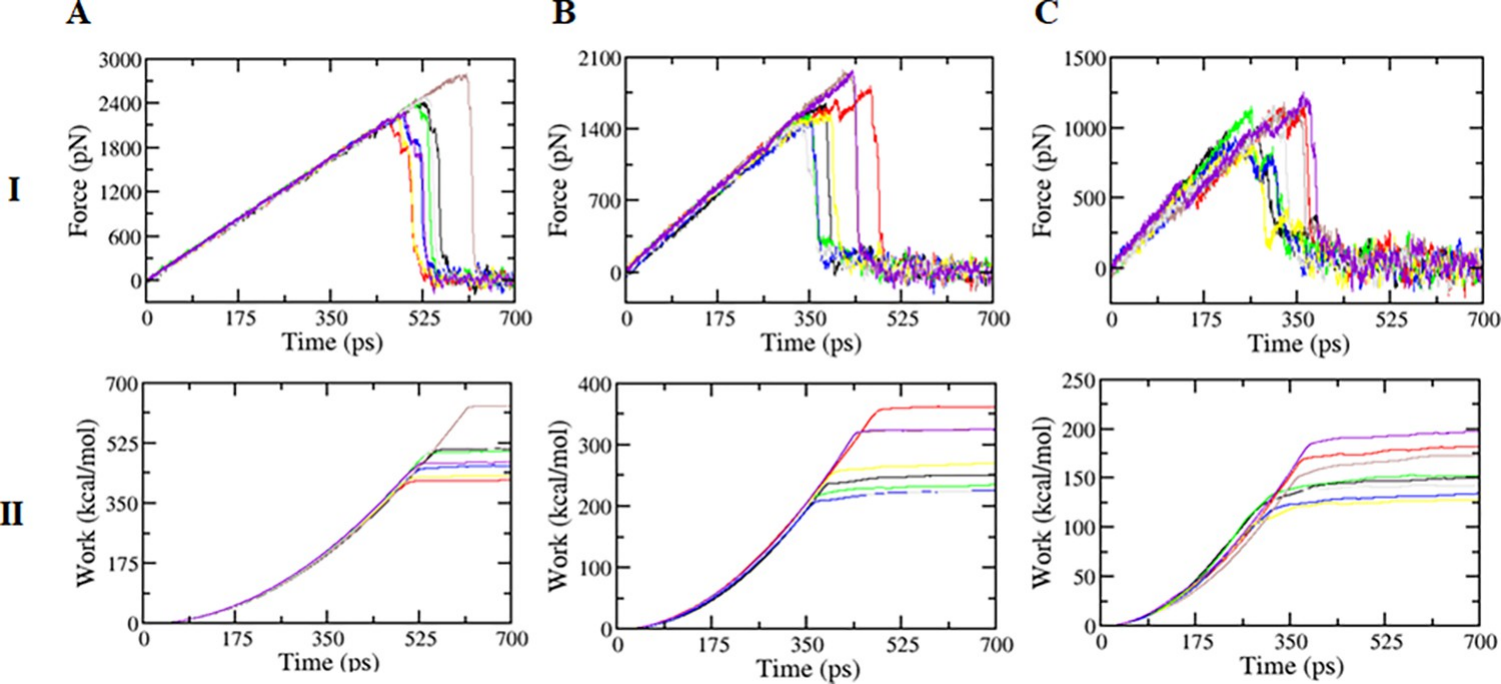
(I) The average values of pulling forces and (II) The work of three complexes in due times of (A) Elagolix, (B) Lobaric acid, and (C) Tetramethrin.

An external force was applied on the ligand to gradually pull the ligand away from the binding site. The magnitude of the force required over time was noted in Fig 6-I. The hmax required for the system to reach the Fmax varies depending on the ligands; and hmax is proportional to the magnitude of Fmax. With the average Fmax of 2399.9, 1686.9, and 1094.7 pN for elagolix, La, and Tm, the hmax fluctuated in the ranges of 450–600, 350–525, 265–400 ps, respectively (Table 1).

The work of the external force obtained from Eq 2 was the integral of the external force acting on the ligands. It could be seen that this work increased steadily with time up to hmax, then it was constant and stable (Fig 6-II). The average magnitude of work of elagolix, La, and Tm was 491.3, 277.5, and 157.7 kcal/mol, respectively (Table 1; S1–S3 Videos).

## Discussion

Many environmental pollutants like organophosphates and pyrethroid are known for their reproductive toxicity that may reduce human’s fertility [32]. However, the mechanisms through which these chemicals introduce toxicity to reproductive organs are not well defined [33, 34]. Assessment of the estrous cycle in rodents is a crucial measure of integrity of the hypothalamic-pituitary-ovarian reproductive axis and it can contribute an important information regarding the nature of a toxicant insult to the reproductive system [35, 36]. Therefore, in the present study, we aimed to investigate the biochemical effects of Tm, a type I synthetic pyrethroid, on ovaries of Wistar rats after long-term exposure as well as the attenuation effects of La in the Tm-exposed rats.

Initially, the estrous cycle of rats in all four groups were similar and normal. Later, Tm-treated female rats showed a significant increase in the length of estrous cycle by due to increase in the length of diestrus phase. The elongation of the diestrus phase during the estrous cycle was previously reported in rats exposed to broad-spectrum insecticides such as fipronil [37], methyl parathion [38] and cypermethrin [39]. Interestingly, the irregularity in estrous cycle of the Tm-treated rats could be abolished with La treatment, at the doses of 50 and 100 mg/kg b.w.

It is well-known that anovulation causes disturbances in the feedback signaling from ovarian hormones to the hypothalamus and pituitary which, in turn, affect the production of GnRH, thereby disturbing the normal release of FSH and LH. In the present study, circulating levels of FSH, LH, and P4 hormones were reduced in Tm-treated rats. The oral supplementation of higher dose (100 mg/kg b.w) of La for 30 days improved serum levels of LH, FSH, and P4 in Tm-treated animals (Fig 3A, 3B and 3D), suggesting the roles of La in retaining LH and FSH hormone levels and thus recuperation normal ovulation in Tm-exposed rats. These data support a possibility that La acts on hypothalamic/pituitary site, and thus affecting steroidogenesis [40] that in turn is accountable for follicular growth, maturation, ovulation, and corpora lutea formation in rat [41].

Additionally, Tm-treated group animals showed a reduction in the weights of ovaries and uterus, as compared to the control group (Fig 2A). Loss of ovarian weight accompanied by anestrous was probably due to alteration in reproductive hormones. A significant decrease in the ovarian [32] and uterine weights [7] were also reported in female rats exposed to organophosphate insecticides and Tm, respectively. However, these negative effects were significantly lessened in (Tm + La) groups (Fig 2A).

Estrogen is chiefly responsible for the regulation of estrous cycle in lower mammals [42], suggesting that the alteration in estrous cycle in the present study may be due to the lower levels of estrogen in Tm-treated animals. Furthermore, the production of E1 and E2 by ovarian cells of the Tm-treated rats was significantly reduced due to the imbalance in T and P4, which ultimately disturbed the estrous cycle in these animals (Fig 3C–3F). Our results concurred with the previous studies which showed a decrease in both serum estradiol levels and its production by ovarian cells in insecticides-exposed female albino rats [27, 34]. Importantly, our study has also indicated that co-treatment of rats with (Tm + La) reversed the serum level of E1 and E2 in Tm-treated animals at a time- and dose-dependent manner (Fig 3E and 3F), possibly via hypothalamic/pituitary axis. It is presumed that effects of La on the ovulatory process was due to its estrogenic property that either activates the estrogen receptors or increases estrogen synthesis by improving LH and/or FSH concentrations.

Finally, the alteration of length of estrous cycles and imbalance in hormonal levels were confirmed by the photomicrograph of vaginal smears of treated rats. The daily vaginal cytology of Tm-treated animals revealed an increase in the estrous cycling duration (from 4 days to 6–7 days) (Fig 2B). Moreover, smears of Tm-treated rats showed the presence of rEC, clumps of NEC and CSEC and rNEC in different phases of the estrous cycle. However, the number of these abnormal cells were reduced in the smears of (Tm + La-L)-treated rats and completely disappeared in the smears of (Tm + La-H)-treated rats signifying a reversal trend of the irregularity in the estrous cycle in La-treated rats (Fig 4).

Our results suggested that Tm inhibits the secretion of LH and FSH by affecting hypothalamic/pituitary axis, which, in turn, reduces the serum level of E1 and E2 in Tm-treated animals. Lower level of E1 and E2 might disturbs the estrous cycle, and consequently interferes with ovaries and uterus by inducing cytological and biochemical modifications in Tm-treated animals. However, these actions were counteracted by administration these Tm-treated rats with La.

To answer the question of “how could the natural compound La counteract the toxic effects of Tm?”, the docking studies were carried out. We hypothesized that Tm binds and inhibits the transmembrane proteins such as GnRH receptor, one of the crucial upstream factors of the sex hormone production cascade. Therefore, to counteract Tm effect, La must exhibit an antagonistic role to this pesticide at the same binding site on the GnRH receptor. In addition, the reference structure of GnRH receptor, 7BR3, was co-crystallized with its antagonist, elagolix [43]. The proven mechanism suggests that elagolix blocks GnRH receptor in the pituitary gland, preventing GnRH-induced secretion of FSH and LH from the anterior pituitary, and thereby reducing production of the gonadal hormones [44]. The docking results revealed that elagolix is the best inhibitor against GnRH receptor, with about 1.5 times higher strength than that of Tm and La. However, Tm and La could compete fairly for the same binding site on the receptor. To check if the binding of La inhibits GnRH receptor completely, a kinetic study of the dissociation of the ligand from the binding site was performed. Briefly, a complete trajectory described from the moment when an external force was applied to the ligand until it reached a maximum and rapidly decreased to a stable value (S1–S3 Videos). The Fmax point could be viewed as equivalent to the transition state of bound and unbound states between ligand and protein. The larger the non-equilibrium work required to achieve this transition state, the higher the energy required to dissociate the ligand from the binding site would be. According to the results, the effect of La on GnRH receptor would be approximately twice as long as Tm but is only half of that of elagolix. This finding might explain why the elimination cycle of La was shorter than elagolix and the production of reproductive hormones was still possible afterwards.

## Conclusions

In conclusion, the current study revealed that Tm exposure significantly altered the estrous cycle, weight of the reproductive organs, and serum levels of hormones in female rats. The alteration of hormonal levels including LH, FSH, T, P4, E1, and E2 might have affected steroidogenesis in the ovary. However, supplementation of La along with Tm reversed serum levels of gonadotropins probably by affecting hypothalamic/pituitary axis. Specifically, La, as an antagonist, had the potential to competitively inhibit Tm binding to GnRH receptor in terms of both kinetic and thermodynamic process but did not cancel the response of GnRH receptor. Hence, La significantly ameliorated the Tm-induced toxic effects. This study also proves the rationale for the traditional usage of *Stereocaulon* and *Usnea* to treat menstrual complaints, vaginal discharge, and swelling of female genitalia.

## Supporting information

S1 Video The steered molecular dynamic process simulates the dissociation of elagolix from the 7BR3 protein. (MP4)

S2 Video The steered molecular dynamic process simulates the dissociation of lobaric acid from the 7BR3 protein. (MP4)

S3 Video The steered molecular dynamic process simulates the dissociation of tetramethrin from the 7BR3 protein. (MP4)
